# Short-Term Effect of Low-Dose Atropine and Hyperopic Defocus on Choroidal Thickness and Axial Length in Young Myopic Adults

**DOI:** 10.1155/2019/4782536

**Published:** 2019-08-21

**Authors:** Beata P. Sander, Michael J. Collins, Scott A. Read

**Affiliations:** Contact Lens and Visual Optics Laboratory, School of Optometry and Vision Science, Queensland University of Technology, Victoria Park Road, Kelvin Grove 4059, Brisbane, Queensland, Australia

## Abstract

**Purpose:**

To examine the interaction between a short period of hyperopic defocus and low-dose atropine upon the choroidal thickness and ocular biometrics of healthy myopic subjects.

**Methods:**

Twenty young adult myopic subjects had subfoveal choroidal thickness (ChT) and ocular biometry measurements taken before and 30 and 60 min following the introduction of optical blur (0.00 D and −3.00 D) combined with administration of 0.01% atropine or placebo. Each combination of optical blur and drug was tested on different days in a fixed order.

**Results:**

The choroid exhibited significant thinning after imposing hyperopic defocus combined with placebo (mean change of −11 ± 2 *μ*m, *p* < 0.001). The combination of hyperopic blur and 0.01% atropine led to a significantly smaller magnitude of subfoveal choroidal thinning (−4 ± 8 *μ*m), compared to placebo and hyperopic defocus (*p* < 0.01). Eyes treated with 0.01% atropine with no defocus exhibited a significant increase in ChT (+6 ± 2 *μ*m, *p* < 0.01). Axial length also underwent small but significant changes after treatment with hyperopic blur and placebo and 0.01% atropine alone (both *p* < 0.01), but of opposite direction to the changes in choroidal thickness. However, the 0.01% atropine/hyperopic blur condition did not lead to a significant change in axial length compared to baseline (*p* > 0.05).

**Conclusion:**

Low-dose atropine does inhibit the short-term effect of hyperopic blur on choroidal thickness and, when used alone, does cause a slight thickening of the choroid in young healthy myopic adults.

## 1. Introduction

Myopia is one of the most common types of refractive error and a leading cause of functional visual loss [1]. Despite extensive attempts to develop effective strategies to combat myopia, there is no fully effective treatment that will prevent its development and progression. Clinical trials examining various myopia control interventions indicate that muscarinic blockers (atropine and pirenzepine) appear to have the strongest preventative effect on myopia progression [2–5]. However, at higher concentrations (above 0.02%), atropine produces ocular side effects such as pupillary dilation, photophobia, and difficulty with near focus (cycloplegia) that limit its practical application [6–10].

As early as mid of 19th century, atropine was proposed as a treatment for myopia control [11], with numerous clinical studies assessing it effectiveness over the past three decades [6, 12, 13]. But it was not until the publication of findings from randomized controlled clinical trials in mainly East Asian children that atropine was recognized as an effective treatment for myopia [7, 8, 10, 14–16]. An important observation from the ATOM 2 study showed that low-dose (0.01%) atropine is almost as effective as higher concentrations (0.5%, 0.25%, and 0.1%) of atropine in slowing the progression of the spherical equivalent refraction (SEQ) of myopia while causing less visual side effects [8]. However, it is worth noting that there was a discrepancy between the refractive error and axial length data for low-dose atropine in this study, with the axial elongation observed in the 0.01% atropine group appearing comparable to that observed in the placebo control group [15]. Although it takes initially longer to produce a therapeutic effect (more than three months), 0.01% atropine yielded a similar reduction in SEQ myopia progression to higher doses in a five-year follow-up study, with a marked reduction in the “rebound effect” that was observed during washout after higher doses [16]. The exact mechanism underlying the “rebound effect” is unclear, but the phenomenon leads to a rapid increase in myopia (0.5 D/year) in children originally treated with higher concentrations of atropine (0.1%, 0.25%, and 0.5%, 1.0%) upon cessation of treatment.

Although much work on the potential of low-dose atropine against myopia has been carried out, there is still considerable ambiguity with regard to its optimal low concentration that is most effective to prevent myopia and its mechanism of action. The current clinical trial (LAMP) has shown the ability of different concentrations of low-dose atropine (0.05%, 0.025%, and 0.01%) to slow myopia progression in myopic children, with 0.05% atropine being the most effective in controlling axial length and SEQ progression [17]. Further, it is generally accepted that atropine inhibition of myopia does not rely on paralysis of accommodation [18] but that atropine may act (directly via a muscarinic mechanism or indirectly through a nonmuscarinic mechanism) on posterior segment tissues such as the retina, retinal pigment epithelium (RPE), choroid, or sclera in order to influence eye growth [19–22]. However, a consistent finding in atropine clinical studies is a reduction in refractive error SEQ progression which is not matched by a reduction in axial length progression, suggesting a possible role for the ciliary muscle in the refractive error changes [7, 8, 10, 14, 16, 17].

Choroidal thickness shows short-term sensitivity to a range of antimuscarinics (atropine, homatropine, and cyclopentolate) that have generally been shown to significantly increase subfoveal choroidal thickness in humans [23–25]. Further, a range of different muscarinic antagonists have also been identified as being able to slow eye growth and trigger a transient thickening of the choroid in animals treated with hyperopic defocus that would typically be expected to lead to choroidal thinning [26, 27]. Recently, two studies have shown that high-dose antimuscarinic agents (atropine 0.5% and homatropine 2%) can inhibit the effect of hyperopic defocus (typically leading to thinning) on subfoveal choroidal thickness [28, 29]. However, the practical question remains whether low-dose atropine (0.01%) can also inhibit short-term changes in choroidal thickness and axial length in response to hyperopic defocus.

In this context, we examined the interaction between short periods of hyperopic retinal defocus and 0.01% atropine upon the choroidal thickness and axial length of young healthy myopes. By investigating ocular changes after combined interventions, we hoped to improve our understanding of the myopigenic mechanisms influencing the thickness of the choroid in humans and provide insights into the possible mechanism underlying the myopia control effects of low-dose atropine.

## 2. Materials and Methods

### 2.1. Subjects

Twenty myopic subjects (spherical equivalent refraction of ≥−0.75 DS) with a mean age (±SD) of 27.3 ± 5 years were recruited primarily from the students and staff of the Queensland University of Technology to participate in this randomized, single-masked, placebo-controlled study. The investigation conformed to the principles outlined in the Declaration of Helsinki. Approval was obtained from the university human research ethics committee, and participants gave their informed consent before the experiment. The sample size used in the study provided 80% power to detect a choroidal thickness change of 11 ± 3 *μ*m, based upon the findings from our previous work [29]. Of the study population, 70% (*n* = 14) were female and 45% were Caucasian (Caucasian *n* = 9, East Asian *n* = 8, Indian *n* = 2, and Middle Eastern *n* = 1).

Ahead of the study, each participant had a full eye examination, and those with serious eye or systemic problems, history of eye trauma or surgery, or any record of previous myopia interventions were excluded from the experiment. All enrolled participants demonstrated good visual acuity of logMAR 0.00 or better and had a range of refractive errors (spherical equivalent from −0.75 to −6.00 DS). The mean spherical equivalent refractive error was −2.87 ± 1.64 DS. During the experiment, care was taken to test each subject at approximately the same time of day between 9 am and 2 pm, to minimize the potential confounding effect of ocular circadian fluctuations in choroidal thickness and axial length upon the results [30]. The experiment trials consisting of a combination of blur (either monocular hyperopic blur (−3 D) or optimal focus) and atropine (one drop of 0.01% atropine) or placebo (0.3% hydroxypropyl methylcellulose) were tested on separate days, in a fixed order. A hyperopic defocus/placebo trial was tested first and was followed by a no defocus/placebo eye drops trial, a hyperopic defocus/0.01% atropine trial, and finally a no defocus/0.01% atropine trial. We decided to use a fixed order design to minimize the possible contamination of subsequent trials due to the residual action of the previously administered atropine. The sessions were spaced at least two days apart with an average time of 49.03 ± 0.6 hr between sessions. This two-day interval was based on a washout period of five to ten times the terminal elimination half-life of the drug [31], and atropine's terminal half-life is 2.5 ± 0.8 hours [32].

### 2.2. Pharmacological Agents

One drop (∼33 *μ*L) of 0.01% atropine (consisting of 0.0005 g of atropine sulphate, 1.405 g of 0.9% sodium chloride, 0.245 g of 0.001% benzalkonium chloride, and 2.8 g of water) or placebo (0.3% hydroxypropyl methylcellulose) was instilled into the right eye, combined with a different blur condition at each visit. The atropine dose of 0.01% was chosen based on the effective dosage and low rate of adverse effects reported in previous randomized, controlled clinical trials [8, 16]. Since 0.01% concentration is thought to be efficacious in myopia control and to have less disruptive effect on the patient daily activities compared with higher doses of atropine, we decided to use it in our study. The 0.01% dose is also predicted to exceed the published ID50 values (concentration that binds 50% of the possible maximum to the target receptor) of atropine [33]. We attempted to mask participants to the pharmacological agent; however, true masking cannot be achieved due to the nature of the drug (e.g., some burning sensation after the atropine administration).

### 2.3. Procedures

All subjects had a set of retinal and choroidal scans as well as ocular biometry collected before and then 30 and 60 min following the start of the trials. To control the potential confounding effect of accommodation on choroidal thickness and axial length results, participants were asked to maintain distance fixation at six meters (watching TV) with their optimal refractive correction for 20 minutes prior to and between measurements. Further, to limit proximal accommodation during biometric measurements, a periscope system was attached to a noncontact biometer (Lenstar LS 900; Haag-Streit AG, Koeniz, Switzerland), as per Sander et al. [23].

The Copernicus SOCT-HR (Optopol Technology S.A., Zawiercie, Poland) was utilized to obtain multiple orthogonal (90- and 180-degree cross pattern), 6 mm length, foveal-centered, chorioretinal B-scans, with each set of scans collected consisting of 30 horizontal and 30 vertical B-scans [29]. Three sets of OCT B-scans were captured from the right eye at baseline (preintervention) and then at 30 and 60 minutes after the introduction of the blur/drug condition and were later averaged.

Ocular biometric data were also measured at the same times using the Lenstar LS 900 biometer [23]. Five separate ocular biometric measurements were acquired for each measurement session, and the data were later averaged.

### 2.4. Data Analysis

Following data acquisition, the individual B-scan images collected at each session were averaged, and the horizontal and vertical OCT images of the retina and choroid were manually segmented by a masked observer, using customized software [34]. The average foveal retinal thickness was calculated as the axial distance between the ILM and the RPE on each scan, while the average subfoveal choroidal thickness was defined as the distance between the outer boundary of the RPE and the inner boundary of the chorioscleral interface at the fovea. The average biometric data from the Lenstar LS900 (axial length, central corneal thickness, anterior chamber depth, and lens thickness) were also analysed for each testing condition.

As data from all variables were normally distributed at each time point, as assessed by the Kolmogorov–Smirnov test of normality (*p* > 0.05), a repeated-measures analysis of variance (ANOVA) that examined the effect of defocus, drug, and time on ocular parameters was then conducted. Each of the measured variables was used to determine the significance of changes in each of the ocular parameters as a result of the interaction between the different blur conditions and pharmacological agents. The Bonferroni-adjusted post hoc analyses were employed to examine the difference in ocular parameters with significant within-subject effects and interactions.

## 3. Results

### 3.1. Within-Session Repeatability

The within-session SD of the ocular biometrics was axial length (11 *μ*m), central corneal thickness (2 *μ*m), anterior chamber depth (12 *μ*m), lens thickness (19 *μ*m), retinal thickness (2 *μ*m), and 3 *μ*m subfoveal choroidal thickness. ICC analysis suggested “excellent” reliability for all variables (ICC > 0.90 for all variables).  Table 1 illustrates the repeatability and reliability data for each of the ocular parameters across all measurement sessions.

### 3.2. Subfoveal Choroidal Thickness

Repeated-measures ANOVA showed a statistically significant increase in subfoveal choroidal thickness from baseline as a result of low-dose atropine, a significant interaction between the effect of low-dose atropine and time, as well as a significant interaction between low-dose atropine, blur condition, and time (all *p* < 0.05).  Table 2 shows the change in subfoveal choroidal thickness for all four conditions tested, in comparison with baseline thickness over 30 and 60 minutes.

The combination of hyperopic blur and low-dose atropine led to a relatively small amount of subfoveal choroidal thinning (mean change: −2 ± 4 *μ*m and −4 ± 8 *μ*m after 30 and 60 minutes, respectively) that was not significantly different to baseline (both *p* > 0.05). However, hyperopic blur and placebo led to a small and statistically significant decrease in subfoveal choroidal thickness (mean change: −6 ± 1 *μ*m, *p*=0.008, and −11 ± 2 *μ*m, *p*=0.0001, compared to baseline after 30 and 60 minutes, respectively), and this magnitude of choroidal thickness change was significantly different to that observed for the low-dose atropine and hyperopic blur condition (*p*=0.019 at 60 minutes). The low-dose atropine with no defocus condition caused a small increase in subfoveal choroidal thickness that was statistically significant at 60 minutes (mean change: +2 ± 1 *μ*m, *p*=0.234, and +6 ± 2 *μ*m, *p*=0.011, at 30 and 60 minutes compared to baseline).

**Figure 1:**
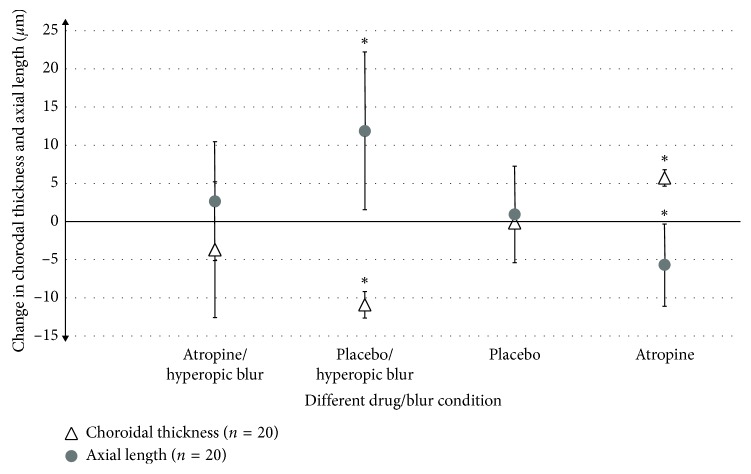
Mean difference in subfoveal choroidal thickness and axial length at 60 minutes after the introduction of the four drug and blur conditions for 20 subjects. Asterisks imply significant differences in choroidal thickness and axial length compared to baseline (*p* < 0.01). Error bars represent ±SD.

No significant change in the subfoveal choroidal thickness was found with the placebo and no defocus (mean change: 0 ± 2 *μ*m and 0 ± 1 *μ*m for 30 and 60 minutes, respectively; *p* > 0.05) (Figure 1). There was also no significant difference between the baseline subfoveal choroidal thickness measurements (prior to drug instillation) for any of the four conditions tested on different days.

### 3.3. Retinal Thickness

All four interventions did not elicit statistically significant changes in retinal thickness at the fovea (Table 2), with the average retinal thickness change being less than 1 *μ*m (*p* > 0.05).

### 3.4. Axial Length

The average ocular biometric changes following the introduction of the four different interventions are illustrated in Table 2 and Figure 1. There was significantly less change from baseline in axial length observed for the low-dose atropine/hyperopic blur condition (+4 ± 8 *μ*m, *p*=0.756, and +3 ± 8 *μ*m, *p*=0.87) compared to the placebo/hyperopic blur (mean change: +6 ± 9 *μ*m, *p*=0.119, and +12 ± 10 *μ*m, *p*=0.006) at 30 and 60 minutes, respectively. Eyes treated with low-dose atropine/no defocus exhibited shortening of the axial length, and this was statistically significant at 60 minutes (mean change: −3 ± 7 *μ*m, *p*=0.356, and −6 ± 5 *μ*m, *p*=0.036, at 30 and 60 minutes).

### 3.5. Anterior Eye Biometry

Low-dose atropine alone elicited changes in anterior segment components, with anterior chamber depth significantly increasing from baseline (average mean change +38 ± 14 *μ*m, *p*=0.023) and crystalline lens thickness significantly decreasing from baseline (average mean change −24 ± 13 *μ*m, *p*=0.044) (Table 2). However, both the placebo/hyperopic blur and the low-dose atropine/hyperopic blur conditions did not cause significant changes in anterior chamber depth or lens thickness (both *p* > 0.05). Central corneal thickness showed no significant changes for any of the tested conditions (all *p* > 0.05).

## 4. Discussion

The current study has demonstrated that 0.01% atropine produces a small increase in subfoveal choroidal thickness. The magnitude of subfoveal choroidal thickness increase with 0.01% atropine (6 *μ*m) was lower than that reported with 1% atropine (15 *μ*m) [25], 2% homatropine (14 *μ*m) [23], and 1% cyclopentolate (21 *μ*m) [24], suggesting a possible dose-dependent response. The inhibition of choroidal thinning with hyperopic defocus by 0.01% atropine is also consistent with earlier studies where muscarinic blockers (0.5% atropine [28] and 2% homatropine [29]) prevented the reduction in choroidal thickness produced by hyperopic blur.

Atropine is a potent muscarinic blocker; however, the exact mechanisms and pathways involved in atropine's antimyopigenic effects as well as site of action for atropine-meditated myopia inhibition are not clear. Drug absorption following topical application to the eye is a complex process that tends to be influenced by drug kinetics in the cul-de-sac of the conjunctiva and corneal permeability. The atropine eye drops used in this study were combined with benzalkonium chloride (BAK) 0.1 mg/mL, which improves penetration through the cornea [35]. Further, once inside the eye, atropine reaches the intraocular concentration of 659 nM, which is significantly higher than IC50 value for atropine (20 nM) for the human iris and ciliary muscle receptor when using carbachol as the agonist [33] and its affinity at human M4 receptor (0.125–0.25 nM) [36]. Therefore, the concentrations of atropine in the eye after a single topical application in this study are likely to be within a range capable of reaching the choroid within 60 minutes.

Muscarinic receptors including M_1_, M_2_, and M_4_ receptors have been implicated in the development and/or progression of myopia in animal models [20, 21, 26, 37]. Therefore, giving atropine's ability to block muscarinic receptors in the posterior segment, it may interfere in the biochemical cascade involved in the transient response to hyperopic blur and thus prevent myopia. It is important to notice, however, that none of the experimental studies has revealed a presence of a direct correlation between muscarinic receptors in the posterior segment and the antimuscarinic properties of atropine for inhibition of myopia. Further, emerging evidence seems to substantiate nonmuscarinic mechanism in antimyopia effects of atropine. Major arguments that contradict cholinergic mechanism are lack of effectiveness of majority of muscarinic antagonists against myopia progression in experimental studies [20], the high tissue concentrations of muscarinic antagonists (above muscarinic receptor affinity constants) required to inhibit myopia in experimental studies [38], and in vitro data supporting nonmuscarinic targets for atropine including nitric oxide, dopamine, or *α*2-adrenoreceptors [36, 39].

Previous experimental studies have shown that atropine may trigger the production and depletion of nitric oxide (NO) and this, in turn, impacts choroidal thickness changes [27, 39]. A suppression of prejunctional M_2_/M_4_ muscarinic receptors on cholinergic-nitrergic nerve terminals in the choroid by atropine modulates a vasodilation response in ocular blood vessels through the neural nitric oxide pathway and this, in turn, influences choroidal thickness changes and ocular growth [40, 41]. Similarly, data of ATOM 2 clinical trial [16] have supported, although indirectly, a nonmuscarinic mechanism. Outcomes of the trial have revealed the development of a “rebound phenomenon” in children who were originally treated with higher concentrations of atropine for 24 months and showed an enhanced myopia progression 12 months after cessation of the therapy. Although the exact mechanism underlying the “rebound effect” is unclear, prior cardiovascular research showed that nitrates, widely used to promote vasodilation via release of nitric oxide, generate a rebound phenomenon. This phenomenon develops when the medication is stopped after continuous use and is probably related to desensitization of the NO-dependent soluble guanylyl cyclase (sGC)/cyclic guanosine monophosphate (cGMP) signalling pathway [42, 43].

Further, some evidence suggests that the ability of atropine to prevent myopia development and/or progression may involve a release of dopamine in the retina, resulting in a transient choroidal thickening and inhibition of ocular growth. Zhong et al. [44] proposed that the eye's response to optical blur is driven by the activity of the amacrine cells. While it has not yet been fully established whether amacrine cells regulate eye growth, previous work has demonstrated that dopaminergic amacrine cells could play an important role in the detection of ocular defocus [45]. Their function is controlled by suppressive muscarinic cholinergic amacrine cells [46] and GABAergic amacrine cells [47]. Therefore, it is possible that atropine interferes with dopaminergic signalling in the retina by influencing the muscarinic cholinergic amacrine cell responses leading to myopia prevention. Previous research showed that muscarinic blockers may stimulate the synthesis and release of dopamine from dopaminergic amacrine cells that eventually cause expansion of the choroid and retardation of ocular growth [26, 48, 49]. Recent work by Khanal et al. [50] provides further evidence that topical atropine may modify inner retinal cell responses, since multifocal ERG changes evident in the presence of myopic defocus were found to increase in magnitude in the inner peripheral retina, following the instillation of topical atropine. The mechanism of how atropine influences inner retinal dopaminergic signalling, however, has not yet been sufficiently clarified. Recently, Carr and colleagues [36] have demonstrated that atropine, like other muscarinic antagonists, binds to *α*2-adrenoreceptors at concentrations similar to those used to suppress experimental myopia in chicks. As adrenoreceptors are known to control the activity of tyrosine hydroxylase, the key enzyme in dopamine synthesis, it is possible that atropine acting on *α*2-adrenoreceptors affects the dopamine level in the retina.

Relatively large magnitude changes were observed in the anterior chamber depth (40 microns deeper) and lens thickness (29 microns thinner) following atropine instillation consistent with a reduction in accommodative tone (Table 2). This supports the possibility that the choroidal thickness changes observed may at least partially be related to the biomechanical forces generated through the relaxation of the ciliary muscle with 0.01% atropine. Previous work shows that changes in accommodation [51] can result in small magnitude choroidal thickness changes.

Similar to previous clinical trials [16, 17], 0.01% atropine, probably due to the minimal magnitude of choroidal thickness changes, did not produce significant changes in axial length. It would be of significant clinical interest to determine if continued treatment with 0.01% atropine leads to a long-term increase in choroidal thickness and thus to a reduction in axial elongation. This, in turn, would decrease the likelihood of developing pathological myopia. The administration of 0.01% atropine also produced an increase in the anterior chamber depth (backward lens movement) and decreased lens thickness, which are both related to the change in ciliary muscle tone and alter the biomechanical forces on the globe.

The study has a number of limitations that need to be considered. The relatively small sample size of 20 subjects is a limitation, along with the 60-minute test duration and the mixed ethnicity of the subjects. Testing over longer durations is difficult because of the need to continuously control the type of visual tasks (accommodation demand) and account for the natural diurnal cycle in choroidal thickness [30, 51]. Testing groups of different ethnicities including East Asians would be useful, since the highest prevalence of myopia occurs in East Asia [52, 53]. Results of a recent systematic review suggested atropine has been more effective in controlling myopia progression in East Asian children compared with Caucasian children [54]. Another shortfall of this study is the relatively small changes in choroidal thickness compared to the measurement accuracy of the OCT. Longer wavelength OCTs and automated segmentation of the choroid should provide more reliable choroidal thickness measurements in the future and will allow better discrimination of small thickness changes. Finally, the use of a single dose of 0.01% atropine (rather than a range of various low concentrations) to assess the short-term ocular changes is another limitation of the study. Recently, Yam et al. [17] have suggested that the higher concentration of low-dose atropine (0.05%) is more effective than 0.01% in controlling SEQ myopic progression and eye growth. However, higher concentrations above 0.02% tend to produce clinically significant pharmacological effects on the iris and ciliary body function. Thus, further work evaluating the effect various concentrations of low-dose atropine on the choroid and eye growth without producing clinically significant side effects is warranted to find the dose that will provide the best balance between benefits and side effects for myopia control.

Low-dose atropine can inhibit the short-term effect of hyperopic blur on choroidal thickness and axial length, similar to higher dose of atropine and homatropine [28, 29]. When administered without blur, low-dose atropine also causes a small magnitude thickening of the choroid in young healthy adult subjects. These findings may improve knowledge about the antimyopia effect of atropine treatments, as well as the possible mechanism underlying eye elongation, and may serve as a base for future studies on the development of new myopia prevention strategies and/or treatment options.

## Figures and Tables

**Table 1:** Outline of within-session repeatability and reliability for each of the variables measured at each measurement session.

	Mean within-session standard deviation	Mean coefficient of variation (%)	ICC
AL (*μ*m)	11	0.05	0.998
CCT (*μ*m)	2	0.42	0.998
ACD (*μ*m)	12	0.37	0.997
LT (*μ*m)	19	0.53	0.995
Subfoveal ChT (*μ*m)	3	1.14	0.995
RT (*μ*m)	2	0.92	0.998

AL: axial length; CCT: central corneal thickness; ACD: anterior chamber depth; LT: lens thickness; ChT: subfoveal choroidal thickness; RT: retinal thickness; AA: amplitude of accommodation.

**Table 2:** Effects of 0.01% atropine and placebo with or without hyperopic defocus on the average change in ocular variables at 30 and 60 minutes from baseline.

	ANOVA						
	Average (SD) difference in ocular parameters data from baseline				*p* value		
	0.01% atropine + hyperopic defocus (*μ*m)	Placebo + hyperopic defocus (*μ*m)	Placebo (*μ*m)	0.01% atropine (*μ*m)	Drug	Drug by time	Drug by time by defocus
*AL*							
30 min	+4 ± 8	+6 ± 9	0 ± 7	−3 ± 7	**0.015**	**0.007**	**0.046**
60 min	+3 ± 8	+12 ± 10^*∗*^	+1 ± 6^*∗*^	−6 ± 5^*∗*^			

*CCT*							
30 min	+1 ± 1	0 ± 1	0 ± 1	0 ± 1	0.686	0.427	0.731
60 min	−1 ± 1	−1 ± 1	−1 ± 1	0 ± 1			

*ACD*							
30 min	+19 ± 35	+5 ± 34	+7 ± 4	+21 ± 39	**0.042**	0.058	0.892
60 min	+39 ± 36^*∗*^	+7 ± 36	+4 ± 4	+40 ± 34^*∗*^			

*LT*							
30 min	−10 ± 34	−6 ± 32	−3 ± 33	−11 ± 33	**0.025**	**0.049**	0.678
60 min	−21 ± 35	−4 ± 30	−5 ± 34	−29 ± 31^*∗*^			

*RT*							
30 min	0 ± 1	0 ± 1	0 ± 1	0 ± 1	0.265	0.766	0.364
60 min	+1 ± 1	+1 ± 1	+1 ± 1	+1 ± 1			

*Subfoveal ChT*							
30 min	−2 ± 5	−6 ± 2	0 ± 2	+2 ± 1	**0.014**	**0.001**	**0.0001**
60 min	−4 ± 8	−11 ± 2^*∗*^	0 ± 1	+6 ± 2^*∗*^			

Statistically significant ANOVA changes (*p* < 0.05) are highlighted in bold. Asterisks imply significant differences in variables compared to baseline, using post hoc analysis with Bonferroni adjustment (*p* < 0.05). Positive values represent an increase in the ocular parameter, while the negative values correspond to a decrease in the ocular parameter. AL: axial length; CCT: central corneal thickness; ACD: anterior chamber depth; LT: lens thickness; RT: retinal thickness; ChT: subfoveal choroidal thickness.

## Data Availability

The data used to support the findings of this study are included within the article.
